# KRAS and NRAS pyrosequencing screening in Tunisian colorectal cancer patients in 2015

**DOI:** 10.1016/j.heliyon.2019.e01330

**Published:** 2019-03-19

**Authors:** Raja Jouini, Marwa Ferchichi, Ehsen BenBrahim, Imen Ayari, Fatma Khanchel, Wafa Koubaa, Olfa Saidi, Riadh Allani, Aschraf Chadli-Debbiche

**Affiliations:** a Pathology Department, Habib Thameur Hospital, Tunis, Tunisia; b University of Medicine, Farhat Hached Campus, Tunis El Manar, Tunisia; c University of Sciences, Farhat Hached Campus, Tunis El Manar, Tunisia; d Public Health Institute, Tunis, Tunisia

**Keywords:** Oncology, Genetics

## Abstract

**Background:**

Mutations in KRAS and NRAS often result in constitutive activation of RAS in the epidermal growth factor receptor (EGFR) signaling pathway. Mutations in KRAS exon 2 (codon 12–13) predict resistance to anti-EGFR targeted therapy in patients with metastatic colorectal carcinoma (mCRC). However, it's currently known that a significant proportion of mCRC have RAS mutations outside KRAS exon 2, particularly in exons 3 and 4 of KRAS and exons 2, 3 and 4 of NRAS. No data about RAS mutations outside KRAS exon 2 are available for Tunisian mCRC. The aim of this study was to analyze RAS, using pyrosequencing, in nine hotspots mutations in Tunisian patients with mCRC.

**Methods:**

A series of 131 mCRC was enrolled. Nine hotspots sites mutations of KRAS and NRAS were analyzed (KRAS: codons 12–13, codons 59–61, codon 117 and codon 146, NRAS: codons 12–13, codon 59, codon 61, codon 117 and codon 146) using Therascreen KRAS and RAS extension pyrosequencing kits.

**Results:**

Analysis was successful in 129 cases (98.5%). Mutations were observed in 97 cases (75.2%) dominated by those in KRAS exon 2 (86.6%). KRAS G12V was the most dominated mutation, observed in 25 cases (25.8%), and followed by KRAS G12S and KRAS G12D, each in 17 cases (17.5%). Mutations outside of KRAS exon 2 presented 13.4% of mutated cases and almost a third (28.8%) of KRAS exon 2 wild type mCRC. Among those, 9 cases (69.3%) carried mutations in NRAS exons 2, 3 and 4 and 4 cases (30.7%) in KRAS exons 3 and 4.

**Conclusions:**

RAS mutations outside exon 2 of KRAS should be included in routine practice, since they predict also response to anti-EGFR. That would make certain these patients benefit from appropriate testing and treatment. In addition unjustified expenses of anti-EGFR targeted therapy could be avoided.

## 1 Introduction

Mutations in KRAS and NRAS often result in constitutive activation of RAS in the epidermal growth factor receptor (EGFR) signaling pathway. Mutations in RAS genes, particularly in exon 2, 3 and 4 of KRAS and NRAS have been identified as predictors of resistance to anti-EGFR targeted therapy in patients with metastatic colorectal cancer (mCRC) [1]. Besides, recent studies suggest that analysis of mutations in KRAS exon 2 and outside should be introduced in routine screening of mCRC based on an adequate testing to make certain that patients who are candidates to anti-EGFR therapy benefit from appropriate treatment [2]. In Tunisia, even if patients with mCRC eligible for anti-EGFR therapy are routinely investigated for RAS mutations, no data about RAS mutations outside of KRAS exon 2 are available. The aim of this work was to study RAS mutations in exon 2 of KRAS and outside of KRAS exon 2 using pyrosequencing in a Tunisian series.

## 2 Material and methods

### 2.1 Material

From June to October 2015, 131 formalin-fixed paraffin-embedded (FFPE) mCRC blocks for KRAS and NRAS screening were prospectively and consecutively collected at the department of pathology of Habib Thameur teaching hospital in Tunis; Tunisia (HTH). Oncologists, from both public and private sectors from main oncology centres in Tunisia, willing to know if their patients with mCRC were eligible or not for an anti-EGFR targeted therapy, were contacted in order to send the FFPE blocks. The choice of the molecular analysis platform of HTH department of pathology was based on the existence of adequate CE-IVD equipments, particularly a pyrosequencer, and a well trained team supported by Qiagen professionals. Well-codified administrative procedures have also been put in place. A trial period of three months, from March to May 2015, consisted in comparing the results of RAS mutations of mCRC cases from HTH to those performed in a certified laboratory in Paris with accredited techniques. At the same time, the department was equipped with sufficient KIT and consumables to guarantee a response time within 15 days. Clinical, epidemiological and prognostic factors, including the following parameters: age, sex, tumor size, tumor localization, TNM stage, degree of differentiation, vascular emboli and perineural invasion; were determined referring to patients' pathological records. The study was approved by institutional ethics committee of Habib Thameur Hospital of Tunis (HTHEC) and consent has been obtained from each patient after full explanation of the purpose and nature of all procedures used.

### 2.2 Genomic DNA extraction

For each sample, 20 sections were used for DNA extraction. To minimize the cross-contamination risk, before each use, the blade was renewed and the microtome was wiped by xylene followed by a DNA decontamination reagent (DNA away^TM^, Thermo Fisher Scientific^TM^). Genomic DNA extraction was performed according to Kit (QIAamp® DNA FFPE tissue, Qiagen, GmbH, Hilden, Germany) manufacturer's handbook. Briefly, sections were deparaffinized using xylene and resuspended in an appropriate amount of tissue lysis buffer and proteinase K, then incubated at 56 °C for 24 h. The entire lysate was transferred to the QIAamp Minelute column. During centrifugation, the DNA binds to the membrane and contaminants flow through. Next, residual contaminants were eliminated with wash steps. After elution buffer addition, a full-speed centrifugation was performed to collect a pure and concentrated DNA. Quality control extraction was performed using a nanodrop (Implen, Thermo Fisher Scientific^TM^).

### 2.3 Mutational status analysis

#### 2.3.1 Polymerase chain reaction and agarose gel electrophoresis

Polymerase Chain Reaction (PCR) and pyrosequencing were performed according to handbooks of Kits' manufacturer (Therascreen KRAS Pyro® Handbook and Therascreen RAS Extension Pyro® V2 Kit Handbook) [3]. Nine hotspot sites mutations of KRAS and NRAS were analyzed in this order: KRAS: codons 12–13, codons 59–61, codon 117 and codon 146, NRAS: codons 12–13, codon 59, codon 61, codon 117 and codon 146. After each pyrosequencing, the mutated samples were excluded and only wild-type samples can be amplified for following sequencing.

PCR was performed in a Life Touch thermal cycler (Bioer Technologies, Hangzhou, China) in 25 μl final volume by mixing 12.5 μl of PyroMark PCR Master Mix, 2×; 2.5 μl of CoralLoad Concentrate, 10×; 1μl of primers mix; 4 μl of water supplied with the kit and 5 μl of DNA extract. An unmethylated wild type genomic DNA, supplied with the kit, was used as a positive control for PCR and sequencing reactions. In addition, a negative control (water) was included in every PCR. PCR thermal program is explained in Table 1. Successful and specific amplification was verified by visualizing PCR product on 1% agarose gel stained with ethidium-bromide.

**Table 1:** Thermal PCR programs (3).

Initial activation step		95 °C for 15 min
42 cycles; 3-steps each	Denaturation	95 °C for 20 sec
	Annealing	53 °C for 30 sec
	Extension	72 °C for 20 sec
Final extension		72 °C for 5 min

#### 2.3.2 Single-stranded DNA template preparation and pyrosequencing

Preparation of template and sequencing reactions were performed according to manufacturer's directions [3]. Biotinylated PCR products were immobilized onto streptavidin-coated beads (Streptavidin Sepharose® High Performance beads, GE Healthcare) by mixing 10 μL of PCR product with 2 μL Streptavidin Sepharose suspension and the appropriate amount of the binding buffer. To remove non-biotinylated DNA strand, samples were sequentially denatured using PyroMark Q24 Vacuum Prep Workstation Tool (Qiagen). Immobilized pure single-stranded DNA was then transferred to a microtiter plate containing 0.8 μL target-specific sequencing primer (100 pmol/L). Required volumes of substrates, enzymes, and nucleotides (Gold Reagent Kit, Qiagen) listed in the pre-run report were dispensed in a clean PyroMark Q24 Cartridge (Qiagen). Real-time sequencing was performed using PyroMark Q24 pyrosequencing instrument and software according to the manufacturer's instructions [3]. The Plug-in Report was used to analyse the run. Specimens with low percentage of mutations were reanalysed. List of mutations covered by these 2 kits, is detailed in Table 2.

**Table 2:** List of mutations covered by Therascreen Kits (3).

Gene	Exon	Codon	Covered mutations
**KRAS**	Exon 2	Codon 12	G12D G12V G12C G12S G12A G12R
		Codon 13	G13D
	Exon 3	Codon 59	A59T A59G
		Codon 61	Q61H Q61L Q61R Q61E
	Exon 4	Codon 117 Codon 146	K117N
			A146T A146P A146V
**NRAS**	Exon 2	Codon 12	G12S G12C G12R G12D G12V G12A
		Codon 13	G13S G13C G13R G13D G13V G13A
	Exon 3	Codon 59	A59T A59G
		Codon 61	Q61K Q61R Q61L Q61H Q61Q Q61E
	Exon 4	Codon 117	K117N
		Codon 146	A146T A146P A146V

### 2.4 Statistical analysis

SPSS software, version 21 was used for data entry. Management and data analysis in this study were made by the R software 3.4.4. Continuous variables were represented as mean ± standard deviation. The comparison of means was performed with ANOVA analysis. Binary variables were described and compared according to the chi-square test.

## 3 Results

The 131 FFPEs included were collected from 75 men (57.3%) and 56 women (42.7%) (SR = 1.4). The mean age of patients was 56.1 ± 12.6 years. Specimens were from primary tumor (93; 71%) and metastasis and local recurrence (18; 13.7%). Molecular analysis was successful in 129 cases (98.5%). The 2 other cases harboured a defective quality DNA. The response time was 14.6 ± 10.7 days. In the 129 successful analysis tests and 97 cases were mutated (75.2%). KRAS exon 2 mutations were observed in 84 cases (86.6%). KRAS G12V was the most dominated mutation observed in 25 cases (25.8%), followed by KRAS G12S and KRAS G12D, each in 17 cases (17.5%), KRAS G12C in 12 cases (12.3%), KRAS G12R in one case (1%). Only G13D was observed in codon 13 of KRAS. This mutation was observed in 12 cases (12.3%). Mutations outside KRAS exon 2, were observed in 13 cases (13.4%), representing almost a third (28.8%) of KRAS exon 2 wild type mCRC. Among those, 9 cases (69.3%) carried mutations in NRAS exons 2, 3 and 4 and 4 cases (30.7%) in KRAS exons 3 and 4. Mutations outside KRAS exon 2 included NRAS Q61H in 3 cases (3%), NRAS G12D and A146T, each in 2 cases (2%). The rest of mutations included KRAS Q61E, KRAS K117N, NRAS G12S, NRAS Q61K, NRAS K117N and NRAS A146T, each in one case (1%).

**Fig. 1:**
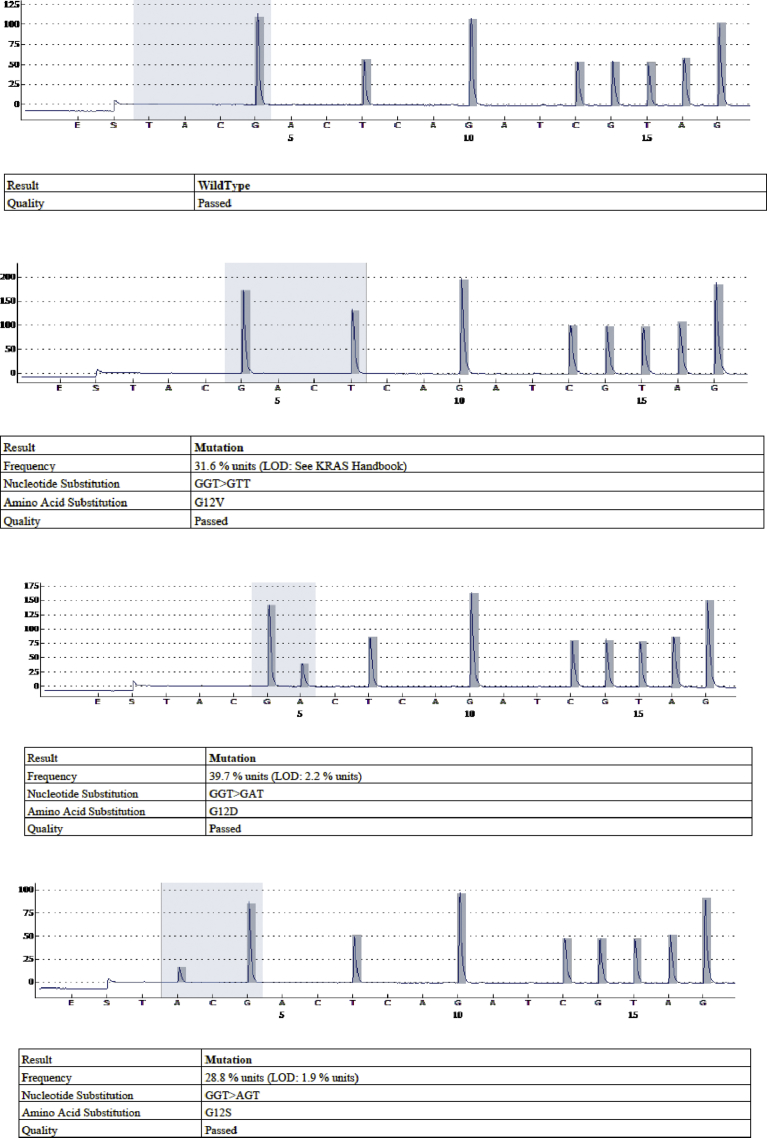
Pyrograms of the wild type and the 3 most predominant mutations in our cohort (from the top: wild type, G12V mutation, G12D mutation and G12S mutation).

Mutation percentage mean was of 21.4 ± 17% (2.3%–99.2%). Mutation percentage median was 15.3% and there were 52 cases (53.6%) with mutation percentage lower than 20%. Mutation classes observed are detailed in Table 3. Fig. 1 illustrates pyrograms of the dominant mutations compared to the wild-type pyrogram. As shown in Table 4, mutation class was significantly associated with degree of differentiation (p = 0.012). The rest of clinicopathological parameters didn't show difference between mutation classes. Thirty two patients with wild type mCRC had benefit from anti-EGFR targeted therapy.

**Table 3:** Mutational status and detailed mutation classes observed.

Mutational status	N = 131 n (%)
Not performed (DNA quality)	2 (1.5)
Successful amplification and sequencing	129 (98.5)
**Wild type**	32 (24.8)
**Mutated**	97 (75.2)
**KRAS exon 2**	84 (86.6) (% of mutated cases)
KRAS, exon 2, codon 12, G12V	25 (25.8)
KRAS, exon 2, codon 12, G12D	17 (17.5)
KRAS, exon 2, codon 12, G12S	17 (17.5)
KRAS, exon 2, codon 12, G12C	12 (12.3)
KRAS, exon 2, codon 12, G12R	1 (1)
KRAS, exon 2, codon 13, G13D	12 (12.3)
**Outside of KRAS exon 2**	13 (13.4)
KRAS, exon 3, codon 61, Q61E	1 (1)
KRAS, exon 4, codon 117, K117N	1 (1)
KRAS, exon 4, codon 146, A146T	2 (2)
NRAS, exon 2, codon 12, G12D	2 (2)
NRAS, exon 2, codon 12, G12S	1 (1)
NRAS, exon 3, codon 61, Q61H	3 (3)
NRAS, exon 3, codon 61, Q61K	1 (1)
NRAS, exon 4, codon 117, K117N	1 (1)
NRAS, exon 4, codon 146, A146T	1 (1)

**Table 4:** Impact of histological type, mutational status and mutation class on clinicopathological parameters.

	Histological type			Mutational status			Mutation class			
	ADK	Mucinous ADK	p	WT	Mutated	p	in KRAS exon 2	outside KRAS exon 2		p
**Sex**										
Male	64 (85.3%)	11 (14.7%)	0.505	22 (29.7%)	52 (70.3%)	0.133	46 (88.5%)	6 (11.5%)	0.562	
Female	50 (89.3%)	6 (10.7%)		10 (18.1%)	45 (81.8%)		38 (84.4%)	7 (15.6%)		
**Resection margins**										
R0	62 (88.6%)	8 (11.4%)	0.109	16 (23.5%)	52 (76.5%)	0.342	44 (86.3%)	7 (13.7%)	0.612	
R1	10 (71.4%)	4 (28.6%)		5 (35.7%)	9 (64.3%)		7 (77.8%)	2 (22.2%)		
**Tumor type**										
Primary	80 (86.0%)	13 (14.0%)	0.745	21 (22.8%)	71 (77.2%)	0.949	61 (87.1%)	9 (12.9%)	0.681	
Metastasis + local recurrence	16 (88.9%)	2 (11.1%)		4 (23.5%)	13 (76.5%)		11 (84.6%)	2 (15.4%)		
**Vascular emboli**										
**+**	46 (78.0%)	13 (22.0%)	**0.029**	12 (21.1%)	45 (78.9%)	0.440	34 (77.3%)	10 (22.7%)	-	
**−**	30 (96.8%)	1 (3.2%)		9 (29.0%)	22 (71.0%)		22	-		
**Differentiation**										
Well				10 (17.5%)	47 (82.5%)	0.304	42 (89.4%)	5 (10.6%)	**0.012**	
Moderately				13 (30.2%)	30 (69.8%)		26 (86.7%)	4 (13.3%)		
Poorly				1 (16.7%)	5 (83.3%)		2 (40.0%)	3 (60.0%)		
**Tumor localization**										
Left colon + rectum	75 (92.6%)	6 (7.4%)	0.022	22 (27.8%)	57 (72.2%)	0.168	49 (86.0%)	8 (14.0%)	0.903	
Right colon	37 (78.7%)	10 (21.3%)		8 (17.0%)	39 (83.0%)		33 (86.8%)	5 (13.2%)		
**T stage**										
T1 + T2	6	-	0.584	1 (16.7%)	5 (83.3%)	0.661	4 (80.0%)	1 (20.0%)	0.552	
T3 + T4	59 (84.3%)	11 (15.7%)		17 (24.6%)	52 (75.4%)		44 (86.3%)	7 (13.7%)		
**N stage**										
N0	25 (86.2%)	4 (13.8%)	0.865	5 (17.9%)	23 (82.1%)	0.414	19 (82.6%)	4 (17.4%)	0.704	
N1 + N2	39 (84.8%)	7 (15.2%)		12 (26.1%°	34 (73.9%)		29 (87.9%)	4 (12.1%)		
**Perineural invasion**										
**+**	39 (84.8%)	7 (15.2%)	0.928	8 (17.8%)	37 (82.2%)	0.171	32 (86.5%)	5 (13.5%)	0.675	
**−**	37 (84.1%)	7 (15.9%)		13 (30.2%)	30 (69.8%)		24 (82.8%)	5 (17.2%)		
**Histological type**										
ADK				27 (24.1%)	85 (75.9%)	0.959	73 (85.9%)	12 (14.1%)	0.582	
Mucinous ADK				4 (23.5%)	13 (76.5%)		11 (91.7%)	1 (8.3%)		
**Age groups**										
<65 years	65 (86.7%)	10 (13.3%)	0.602	15 (20.5%)	58 (79.5%)	0.201	48 (84.2%)	9 (15.8%)	0.481	
≥ 65 years	28 (90.3%)	3 (9.7%)		10 (32.3%)	21 (67.7%)		19 (90.5%)	2 (9.5%)		
**Mutation percentage** (mean ± STD, %)	22.0 ± 18.0	18.7 ± 11.9	0.551							
**Tumor size** (mean ± STD, cm)	3.9 ± 2.0	3.5 ± 2.2	0.529	3.4 ± 2.0	4.0 ± 2.1	0.234	4 ± 2	4 ± 2.3	0.994	
**Age** (mean ± STD, years)	56.0 ± 12.8	57.1 ± 11.6	0.764	55.7 ± 12.7	56.4 ± 12.7	0.806	56 ± 13	55.9 ± 12	0.870	

## 4 Discussion

In our study, KRAS exon 2 mutations were identified in 84 out of 129 tumor specimen (86.6%). Mutations outside KRAS exon 2 were found in 13 cases (13.4%). These latter represented almost a third (28.8%) of wild type KRAS exon 2 mCRC. Mutations outside KRAS exon 2 were identified in exons 3 and 4 of KRAS and exons 2, 3 and 4 of NRAS. NRAS mutations were observed in 6.9% of the 129 specimens. These findings are in line with literature data where mutations of KRAS exon 2 are the most common mutations in mCRC with a frequency reaching up 32–66% [4, 5, 6, 7, 8, 9, 10]. Mutations outside of KRAS exon 2 are found with lower frequency and account for 3%–31% [5, 6, 7, 8, 9, 10, 11, 12]. NRAS mutations are found in 3–22% [5, 7, 8, 9]. It's well known that exon 2 (codons 12–13) of KRAS mutations interfere with anti-EGFR therapy [4] and patients having KRAS exon 2 wild-type mCRC are eligible for anti-EGFR targeted therapy. Nevertheless, more than half of the KRAS exon 2 wild-type mCRC patients don't benefit from this treatment because of resistance to anti-EGFR therapy [13]. It has been recently shown that mutations outside of KRAS exon 2 (exons 3 and 4 of KRAS and exons 2, 3 and 4 of NRAS) can also predict anti-EGFR monoclonal antibody resistance. These mutations called also ‘rare mutations’ have to be included in routine practice, in addition to the previously recommended testing of KRAS exon 2 (codons 12 and 13) before any treatment with anti–EGFR antibody therapy in patients with mCRC [1, 2, 14, 15, 16]. In our study, unjustified targeted therapy with anti-EGFR was avoided in about one third of patients carrying wild type exon 2 of KRAS.

Different techniques are used to assess RAS mutational status. The choice of the most cost-effective method for somatic tumor mutations detection, including KRAS, is a major challenge for molecular pathology laboratories. A number of alternative technologies based on PCR: PCR variants, DNA microarrays, pyrosequencing and next-generation sequencing, etc. were developed to increase mutational analyses sensitivity. These techniques allow the investigation of low enriched tumor samples below the detection threshold of Sanger sequencing of at least 20% [17, 18, 19, 20]. Pyrosequencing is an approach of choice in RAS mutations routine detection. The Therascreen kit is highly sensitive assay able to detect KRAS mutations when they represent as low as 1% of the total DNA [17, 21]. In Tunisia, a PCR-SSCP (Single-Strand Conformation Polymorphism) analysis, confirmed by sequencing in 167 mCRC, has detected KRAS mutations in 31.1% dominated by codons 12 and 13 mutations [22]. In another study using also Sanger sequencing, 31.5 % of 51 mCRC from Tunisian patients harboured KRAS mutations in codons 12 and 13 [23, 24]. In other Tunisian studies, mutation frequency in codons 12 and 13 was ranging from 15% to 46% [25, 26, 27, 28]. In our study, we found G12V the most frequent mutation unlike other studies where G12D was the most frequent one [22, 23]. Different spectrum for the most frequent mutation was reported: G12C [25], G12 S [26], G12D and G13 D [28]. Our study was the first one in Tunisia using pyrosequencing technology in RAS test. This could explain differences observed in mutations frequencies with other Tunisian studies. When cases with mutation percentage lower than 20% and RAS mutations outside of KRAS exon 2 were discarded, the percentage of mutated cases became 31.7% (41 cases). So we think that RAS mutations in mCRC Tunisian patients were underestimated. In a low income country like Tunisia, high-sensitivity techniques should be used to enable the identification of RAS mutations related to targeted therapy resistance. Molecular analyses should include also RAS mutations outside exon 2 of KRAS. Thus unjustified expenses of anti-EGFR targeted therapy could be avoided especially, with the significant increase in the incidence of mCRC in Tunisia (4.5% every year from 1994) [29]. Va devenir num 29 et la reference 29 a sauté.

Moreover, our study included the largest Tunisian series of mCRC and tumor samples were collected from almost all regions in the country. To our knowledge, during the period of the study, all cases of mCRC has been analysed for RAS in our laboratory. These aspects should make our data more representative of the Tunisian population than the other local data.

This study had some limitations which have to be pointed out. The relatively small sample size didn't allow us to make firm conclusions. The missing data didn't allow us to perform a strong statistical analysis. The follow-up wasn't available to perform a prognostic analysis.

In addition, BRAF mutations, suggested to be associated with poor or no benefit from anti-EGFR therapy (oncologist july 2017,864-72), were not analysed in our series and should be performed in a further study.

## 5 Conclusions

This study pointed out that 75.2% of Tunisian patients with mCRC harboured mutations in RAS. Mutations outside of KRAS exon 2 were observed in 13.4% cases. We conclude thereby that RAS mutations in Tunisia were underestimated in previous local studies. We recommend that exons 3 and 4 of KRAS and exons 2, 3 and 4 of NRAS should be henceforth screened in mCRC by using sensitive techniques in order to avoid unjustified treatment and expenses.

## Declarations

### Author contribution statement

Raja Jouini: Conceived and designed the experiments; Performed the experiments; Wrote the paper.

Marwa Ferchichi: Performed the experiments; Wrote the paper.

Ehsen BenBrahim, Imen Ayari: Performed the experiments.

Fatma Khanchel, Wafa Koubaa: Contributed reagents, materials, analysis tools or data.

Olfa Saidi, Riadh Allani: Analyzed and interpreted the data.

Aschraf Chadli-Debbiche: Conceived and designed the experiments.

### Funding statement

This research did not receive any specific grant from funding agencies in the public, commercial, or not-for-profit sectors.

### Competing interest statement

The authors declare no conflict of interest.

### Additional information

No additional information is available for this paper.
